# Measuring Equine-Assisted Therapy: Validation and Confirmatory Factor Analysis of an ICF-Based Standardized Assessment-Tool

**DOI:** 10.3390/ijerph19052738

**Published:** 2022-02-26

**Authors:** Isabel Stolz, Volker Anneken, Ingo Froböse

**Affiliations:** 1 Research Institute for Inclusion through Physical Activity and Sport, German Sport University Cologne, 50226 Frechen, Germany; anneken@fi-bs.de; 2 Research Institute of Movement and Neurosciences, German Sport University Cologne, 50933 Cologne, Germany; 3 Institute of Health Promotion and Clinical Movement Science, German Sport University, 50933 Cologne, Germany; froboese@dshs-koeln.de

**Keywords:** (outcome) assessment, factor analysis, ICF, quantitative research, rehabilitation, therapy evaluation, validation

## Abstract

The International Classification of Functioning, Disability, and Health (ICF) of the World Health Organization (WHO) was established as an international framework for monitoring rehabilitation outcomes and the impacts of health interventions since, as the term “functioning” implies, it emphasizes a person’s “lived health” in addition to their biological health status. Equine-assisted therapy (EAT) represents a holistic intervention approach that aims to improve both biomedical functioning and the patient’s lived health in relation to performing activities and participating in social situations. In this study, the psychometric properties of an ICF-based digital assessment tool for the measurement of the rehabilitation impacts of EAT were analyzed via simultaneous confirmatory factor analyses (CFA) and reliability and sensitivity tests. In total, 265 patients from equine-assisted therapy centers in Germany were included for CFA. Change sensitivity was assessed via multi-level analyses based on 876 repeated assessments by 30 therapists. Results show satisfactory model-fit statistics; McDonald’s omega (ML) showed excellent scores for the total scale (ω = 0.96) and three subscales (ω = 0.95; ω = 0.95, ω = 0.93). The tool proved itself to be change sensitive and reliable (change sensitivity *p ≤* 0.001), retest r = 0.745 **, *p* ≤ 0.001). Overall, the developed assessment tool satisfactorily fulfills psychometric requirements and can be applied in therapeutic practice.

## 1. Introduction

In international health systems, the International Classification of Functioning, Disability, and Health (ICF) of the World Health Organization (WHO) plays a key role in patient recovery. The ICF has widely been established as an international standard for describing a person’s health status and for monitoring the impacts of health interventions to ensure favorable health and rehabilitation outcomes, since it unites biomedical and “lived health” perspectives on functioning and health status [1,2]. Operationalized via the biopsychosocial approach, a person’s functioning can be evaluated by the dynamic interaction between biological aspects, activities, and participation, as well as individual environmental and personal factors. Therefore, a holistic view of an individual’s functioning status in rehabilitation contributes to monitoring the responses of health systems more precisely in relation to the suitability of said responses to their individual health needs [2]. Furthermore, in the WHO global health system, functioning has been considered a third health indicator, alongside mortality and morbidity, and has further been designated the key indicator for rehabilitation [2]. A global aim of successful health strategies has been the reduction in morbidity and mortality and the promotion and assurance of optimal functioning [2]. Monitoring the performance and outcomes of rehabilitation interventions and health services via the functioning indicator will be linkable to the ICF classification, as well as the upcoming ICD-11; therefore, the application and development of ICF-based assessment tools represent a promising method to reduce the complexity of the ICF and create precisely tailored therapeutic interventions in rehabilitation systems [2].

The development and use of standardized and valid assessment in equine-assisted therapy (EAT) has been a huge challenge in the scientific discourse in the past years. Scientific studies have identified EAT outcomes and effects in recent years, yet the absence of both common and consistent terminology usage and clarity in intervention targets and intended therapy outcomes remains a major challenge in the professionalization of the field [3]. EAT represents a holistic intervention approach through horse use, which aims to improve both biomedical functioning and the patient’s lived health in relation to their physical and mental abilities to perform activities and participate in social situations. The umbrella term EAT includes various subdisciplines of equine-assisted interventions, of which the most salient are EAT as curative education in individual and group settings and hippotherapy, a horse-assisted form of physical therapy [3]. Additionally, equine-assisted psychotherapy, trauma pedagogy, ergotherapy, and sports-related interventions represent other areas of equine-assisted therapy and support [3].

A comparison and collection of EAT findings has been complicated in the past years, not only across languages and countries, but also with respect to the reported conditions or intended outcomes of the target groups of EAT and its subdisciplines. As Wood et al. describe in their terminology consensus report, 78 scientific studies could be found that used the term “hippotherapy” in over 60 different ways to describe varying therapy contents and outcomes [4]. Besides restraining progress in the collection of scientific evidence about factors that influence the effects of EAT, this conceptual uncertainty poses a further practical difficulty in the form of reimbursement obstacles with stakeholders, for whom therapy orientations and outcomes might appear opaque [4]. 

In this regard, more accurately assessing EAT and its subdisciplines in intervention practice could be a relevant step within the field of therapeutic subdisciplines, but the challenge of making EAT rehabilitation outcomes comparable with the other health services and interventions within the global health care system must also be considered. To reliably monitor EAT intervention outcomes and validly assess whether rehabilitation goals have been attained in a manner that combines both biological and lived health, the linking of EAT to the ICF classification represents a promising approach to building the basis for systematic and standardized assessment in EAT. Since EAT goals are closely related to rehabilitation targets in terms of functioning, as represented in the ICF via the biopsychosocial view of the patient’s health, the incorporation of EAT outcomes and factors affecting these outcomes could provide an important step in collecting and comparing evidence related to EAT interventions. Furthermore, linking EAT to the ICF classification could provide increased transparency for funding agencies and stakeholders by verifying and validating the effectiveness of therapy outcomes relative to other health care interventions. 

In the past, a few studies have clinically tested the applicability of EAT interventions to the ICF and shown promising tendencies [5,6,7]. In a study by Hsieh et al., the authors concluded that their ICF-CY (ICF children and youth version) assessment approach provided a suitable framework to identify the physical benefits of hippotherapy for children with cerebral palsy (N = 14) [6]. The findings of Borino et al. confirmed the suitability of a self-developed ICF-based assessment tool for measuring behavioral changes and treatment effects in persons with intellectual disabilities participating in EAT and onotherapy (therapy with donkeys) (N = 23) [5]. Authors highlighted the suitability of the ICF-based assessment in terms of quantification of therapy effects, individual treatment planning, and the direct availability of health-related intervention outcomes to the international scientific community [5]. Lanning et al. conducted both two standardized ICF-based questionnaires (the WHODAS 2.0 and the SF36v2) and an ICF-linked qualitative interview to measure the effects of EAT on veterans with post-traumatic stress disorder (PTSD) (N = 51) [7]. The results indicated that the usage of the ICF provided a comprehensive view of the overall functioning of PTSD-diagnosed individuals, examining changes on both mental and physical levels, including dynamic intervention outcomes, and influencing factors, such as environmental aspects and patients’ current health status [7]. The authors emphasize the connection of these health states and physical and social environments in regard to their impacts on an individual’s activities and participation domains, and stress the importance of not overlooking these factors when diagnosing and treating persons with reactive disorders such as PTSD [7].

Considering the promising tendencies of past studies, the aim of this study, realized by the Research Institute for Inclusion through Physical Activity and Sport, was the validation and confirmation of the multidimensional factor structure of an ICF-based standardized assessment tool for the measurement of functioning in EAT and its subdisciplines using the global language of the ICF. For this purpose, an ICF-based assessment tool was developed through an extensive scientific process, field tested in therapeutic practice, and analyzed with regard to its psychometric properties (including both EAT in general and the main subdisciplines of individual EAT, group EAT, and hippotherapy). 

## 2. Materials and Methods

### 2.1. Study Design and Setting

The study employed a longitudinal design. The digital ICF-based assessment tool was tested in therapeutic practice by 30 therapists, who assessed 265 patients with indications for EAT nationwide in 26 EAT centers in Germany. The data collection took place from August 2020 to August 2021 (12 months). The collection period was extended from the originally planned eight-month period to twelve months due to restrictions caused by the COVID-19 pandemic. Therapists also assessed the therapy progress of 127 of these patients with the digital assessment tool over 15 weeks (876 repeated assessments in total). Furthermore, within two additional datasets, therapists assessed retest reliability with a one-week interval and interrater reliability through three repeated measures of the same patients, assessed by three raters. 

### 2.2. Participants

Patients were included in the study if they had an indication for EAT and gave written informed consent (for children, this included the consent of parents or legal guardians). A medical declaration of no objection was conducted to carry out the therapy. Therapies at all involved centers were conducted by professionals according to the standardized nationwide procedure regulations of the German Curatorship for Therapeutic Riding, which additionally ensured that no contraindications were present [8]. The study was approved by the ethics committee of the German Sport University Cologne and in accordance with the 1964 Helsinki Declaration and its later amendments (ethical approval code 076-2019).

### 2.3. Measures

The ICF-based assessment tool was administered using Questback UniPark (Questback GmbH, Cologne, Germany). It comprises a general module for the assessment of functioning in EAT overall and three specialized submodules for the assessment of main EAT subdisciplines (EAT in the individual setting, EAT in the group setting, and hippotherapy). The assessment tool was developed in a preliminary study, where a pilot tool was developed based on qualitative focus group findings, which was in turn linked to the ICF via Cieza’s redefined linking rules [9]. Afterwards, it was field tested and modified after performing exploratory factor analyses and bivariate correlations. Thereafter, the assessment tool was reintroduced to therapeutic practice and evaluated relative to its psychometric properties in this study. The complete tool can be found in Appendix B with the associated ICF code and descriptive statistics (Table A5 general EAT module and all submodules). It is differentiated in a general module, which is to be used superordinated for EAT, as well as the three specified submodules: EAT in the individual and group settings and hippotherapy, and is assessed using a unipolar ten-step Likert scale from “does not apply at all” to “applies fully”. Only endpoint categories were verbalized. The response scaling was designed in this way in order to assess functioning in EAT with a high degree of change sensitivity.

The general module contains 25 items, differentiated in three subscales (motor functioning, mental functioning, and psychosocial functioning). One example item, related to the mental functioning scale of the general module is *G17. Can memorize processes and tasks in the therapy and reproduce them later*, which is linked to the ICF code b1442 Retrieval of Memory. The submodule for EAT in the individual setting contains 11 items in total, differentiated in two subscales (specific motor functioning and specific mental functioning). An example item is *IS05. Is able to adapt their movements to the movements of the horse in a targeted manner*, linked to ICF code b1471 Quality of psychomotor functions of the specific motor functioning scale. The submodule for EAT in the group setting also contains 11 items, differentiated in two subscales (interpersonal functioning and intrapersonal functioning). One example item is *GS06. Can handle conflict constructively*, linked to ICF code d7103 Criticism in relationships, assigned to the interpersonal functioning scale. The hippotherapy submodule contains 16 items, which are differentiated in two subscales (movement functioning and motor control functioning). An example item for submodule H is *H04. Can perceive proprioceptive stimuli (this includes, for example, the perception of movement and position),* which is coded with ICF b260 Proprioception function. It is part of the motor control functioning scale.

All therapists evaluated both the general module and one specified submodule for their patients. In addition, demographic data (gender, age, disability, or chronic disease) were obtained. 

### 2.4. Statistical Analyses

Statistical analyses were performed using the statistical software programs IBM SPSS 27 (IBM Corp, Armonk, NY, USA) and IBM SPSS AMOS 26 (IBM Corp, Armonk, NY, USA). Descriptive statistics of the general module and submodules were calculated (frequencies, means, ± standard deviation). To determine the dimensionality and model fit of the conceptual model, confirmatory factor analyses (CFA) were carried out using the sample covariance matrix. Factorial validity was analyzed using maximum likelihood (ML) analysis. Global fit indices (χ²-Goodness-of-Fit-Test, number of degrees of freedom (df), chi-square fit statistics/degree of freedom (PCMIN/DF), comparative fit index (CFI), root mean square error of approximation (RMSEA), Akaike information criterion (AIC), consistent Akaike information criterion (CAIC), and modification indices (MI)) were examined. As stated by Sherer et al., CFI levels greater than 0.90 were considered to be acceptable, while levels greater than 0.95 were considered to represent a very good fit [10]. For RMSEA, levels of less than 0.08 indicated satisfactory model fit, whereas levels of <0.05 were considered to be a very good fit [10]. AIC and CAIC were used in the fitting process as cutoff values indicating increased model fit. Accordingly, lower AIC and CAIC values indicated increased model fit of the models compared in the fitting process [10]. Construct validity was examined via Cronbach’s alpha (α) and McDonald’s omega (ω). Scales were considered reliable with values of α/ω = 0.70 and α/ω = 0.80 [11,12]. Sensitivity was determined based on an aggregated dataset via repeated measurements over 15 weeks, which were analyzed using hierarchical linear mixed models (GLMM, multi-level analyses). Test stability was assessed via retest reliability and inter-rater reliability on the basis of two additional datasets. Retest reliability was assessed with a one-week interval and analyzed via Pearson correlations. Values greater than 0.7 were considered acceptable, and values greater than 0.8 were considered good [13]. Inter-rater reliability was assessed via intraclass correlations (ICC) of three repeated measures of the same patients with equal intervals, each of which was evaluated by three therapists. Interclass correlations values over 0.6 were considered good and values over 0.75 were considered as very good [14]. Because of the small subsample size, normality could not be assured in this test, therefore results of ICC were examined by the nonparametric Friedman test with Bonferroni adjustment, since the assumptions of repeated-measures ANOVA were not met.

## 3. Results

The sample included 265 patients in total (men = 119, women = 145, other = 1). Of these patients, 55 were adults (>18 years) and 209 were children (not specified: 1). Disabilities were heterogenous and mainly located in areas of motor development and mental-perceptual impairments, such as autism, attention deficit hyperactivity disorders, trisomy 21, or cerebral movement disorders and chronic degenerative diseases such as multiple sclerosis. In addition, psychological diagnoses such as dissociative disorders and posttraumatic stress disorder were included but were uncommon among participants. For the submodule EAT in the individual setting, a total of 115 patients were analyzed, for the submodule EAT in the group setting, a total of 87 patients were assessed, and for the submodule hippotherapy, a total of 60 patients could be included (descriptive data and the correspondence of all items to the ICF classification can be obtained in Table A5 of the Appendix B). 

### 3.1. General Module (G)

For the general module, a conceptual three-dimensional model was developed, based on the results of a preliminary explorative factor analysis (EFA) based on a different sample. Confirmatory factor analysis (CFA) was performed to determine whether the proposed multidimensional three-factor structure of the EFA (Scale 1: Motor functioning, Scale 2: Mental functioning, Scale 3: Psychosocial functioning) fits the data. As Table 1 and Table A1 (Appendix A) indicate, the three-factor structure of the hypothesized model represents an adequate fit for the data, which could be optimized via reduction in items and by allowing cross-loadings and error correlations. Factor loadings of the hypothesized model were acceptable according to the usual criteria, but global fit statistics needed modification [15].

#### 3.1.1. Hypothesized Model (Model 1) Module G

**Table 1:** AMOS Output for Hypothesized Model: Goodness-of-Fit Statistics.

Baseline Comparisons				
RMSEA				
Model	CFI	RMSEA	LO 90	HI 90
Default model	0.810	0.122	0.116	0.127
Saturated model	1.000			
Independence model	0.000	0.269	0.264	0.274
**AIC**				
**Model**	**AIC**	**BCC**	**BIC**	**CAIC**
Default model	2102.634	2119.398	2328.157	2391.157
Saturated model	930.000	1053.734	2594.574	3059.574
Independence model	8801.598	8809.580	8908.989	8938.989

#### 3.1.2. Final Model (Model 14) Module G

In the first step, Item G1 was removed (Model 2), because of cross-loadings with the mental functioning and psychosocial functioning scales (G1 <--- Mental functioning MI = 45; G1 <--- Psychosocial functioning MI= 46). Factor loadings remain robust. Fit indices improved slightly (see Model 2, Table A1, Appendix A). Modification indices showed a residual correlation between Items G12 and G13 (e13 <--> e14 MI = 56) because both items thematize the concept of “trust” as a psychosocial aspect. As such, an error correlation was added and Model 3 was run. The global model fit of Model 3 increased, especially chi-squared (χ² = 1675.2). Modification indices indicate that the error covariance related to Items G23 and G24 (e4 <--> e5 MI = 73) remains a strong misspecified parameter. Both Items thematize different aspects of physical movement on horseback while in motion, so both Items were retained, an error correlation was added, and Model 4 was run. Model fit of Model 4 increased slightly, while modification indices showed high cross-loadings of Item G14 with the mental functioning (MI = 52) and psychosocial functioning scales (MI = 50). Item G14 was therefore reduced to clearly distinguish the scales. Model 5 indicated an improved model fit, while modification indices showed a high cross loading of Item G11 with the mental functioning scale (Item G11 <--- Mental functioning MI= 65); therefore, Item G11 was reduced, and the model run again. Model 6 modification indices showed a residual correlation for Items G21 and G22 (e2 <--> e3 MI = 43), both of which thematize motor control functioning; therefore, an error correlation was added. Model 7 made further progress in the global model fit (CFI 0.875), but still needed improvement for a suitable data fit. Modification indices showed a high residual correlation for Items G16 and G17 (e26 <--> e27 MI = 36), since both items thematize aspects of memory functions. An error correlation was added. Model 8 showed a noticeable improvement in model fit (CFI = 0.880) and modification indices showed a residual correlation of Item G2 with the motor functioning scale (e22 <--- Motor functioning scale MI= 28). Item G2 did not discriminate precisely between the scales, so Item G2 was reduced. 

Model 9 showed a cross-loading of Item G18 with the psychosocial functioning scale (e28 <--- Psychosocial functioning scale), so Item G18 was reduced to ensure a consistent fitting process. Model 10 showed a lower value in the AIC model fit (1073.32) and modification indices showed a residual correlation for Items G21 and G22 (correlated error e3 <--> e4 MI = 26.8), so an error correlation was added again. Model 11 indicated an improved global model fit. Modification indices showed a residual correlation for Items G24 and G25 (correlated error e5 <--> e6 MI = 22.2), both of which operationalize aspects of physical rhythmizing, so an error correlation was added again. In Model 12, fit indices improved slightly, but modification indices showed a residual correlation for Items G23 and G25 (correlated error e4 <--> e6), both of which thematize aspects of balance and physical rhythmizing, so another error correlation was added. In Model 13, global model fit further increased (CFI = 0.910 is acceptable). Modification indices showed a residual correlation of Items G15 and G16 (correlated error e25 <--> e26 MI = 21), which thematize mental functions of consideration and concentration, and thus an error correlation was added. The overall model fit of Model 14 is acceptable and represents the best-fitting model, including all parameters that are meaningful and relevant (χ² = 823.9, df = 264, CFI ≤ 0.90, RMSEA = 0.090, AIC = 945.93, CAIC = 1225.3, Table 2). Modification indices do not show remaining misspecified parameters. In total, five items were reduced via the fitting process to ensure scale economy of the general EAT module. Scale 1 (Motor functions) contains 10 items, Scale 2 (Mental functions) contains 7 items, and Scale 3 (Psychosocial functions) contains 8 items.

The reliabilities of the individual scales are in very good range (α = 0.95; α = 0.95; α = 0.93), while the reliability of the total scale of the general module is in excellent range α = 0.96. Due to the high reliability, a reduction in the instrument was possible for improved temporal–economic implementation. McDonald’s omega (ML) showed higher scores for the total scale (ω = 0.96) motor functioning scale (ω = 0.95), mental functioning scale (ω = 0.95), and psychosocial functioning scale (ω = 0.93), and incorporated the loadings and error correlations of the model (Brown, 2015) [15]. 

For retest reliability, Pearson correlations showed the test stability of the total scale and all subscales: total scale r = 0.745 **, *p* < 0.001; motor functioning scale r = 0.678 **, *p* < 0.001, mental functioning scale r= 0.578 **, *p* < 0.001, psychosocial functioning scale r= 0.622 **, *p* < 0.001; (*N* = 71). Normality and linearity were tested via Q-Q diagrams and scatter plots. 

For the measurement of inter-rater reliability, ten patients were assessed three times each by three independent raters. Intraclass correlation (ICC) is reported throughout the results section with the average values, not individual measures. ICC for the total scale showed significant values over time, which remained robust for all raters (measurement time 1: ICC = 0.788, α = 0.791, *p* = 0.002; measurement time 2: ICC = 0.775, α = 0.786, *p* = 0.003, measurement time 3: ICC = 0.811, α = 0.826, *p* = 0.001). The non-parametric Friedman test with Bonferroni adjustment confirmed significant values concerning the three measurement times; accordingly, patients improve significantly from one measurement time to the next for all raters (Friedman Test: Chi-Square (2) = 7.800, *p* = 0.020, n = 10). Pairwise comparison shows significant changes between the first and the third measurement over time (*p* = 0.007). Between the first and the second measurement, as well as between the second and third measurement, the values show smaller changes (*p* = 0.044, *p* = 0.502). The ranks show steady progress from the first to the third measurement (first measurement: mean rank = 1.30, second measurement: mean rank = 2.20, third measurement mean rank = 2.50). Descriptively, the first and second measurement ranks marks the largest difference. With regard to inter-rater agreement, intraclass correlations did not show significant values, i.e., all raters measure a change, but this change is not measured in a consistent way (ICC = 0.161, *p* = 0.352). 

With respect to change sensitivity, Table 3 and Table 4 show a significant positive change over 15 weeks of therapy for the general module (total scale *p* < 0.001; subscales *p* < 0.001, *p* < 0.001, *p* = 0.007). An aggregated dataset based on repeated measurements over 15 weeks was analyzed using hierarchical linear mixed models (GLMM) to generate meaningful results. The results confirm that the general module sensitively depicts change in patient functioning over the course of therapy. The normal distribution test of the residuals confirms this effect for all scales.

To locate the therapeutic effects exactly over the course of 15 weeks, an additional specific mixed linear model with “time” as a categorical variable was calculated. In this model, each measurement time point was compared to the remaining points, so as to show where the most significant changes were located. This cannot indicate significant treatment effects over time as precisely as the multilevel analysis presented in Table 3, Table 4 and Table 5, so it is only used for additional information. The model located the main therapy effects in the first three therapy weeks (week 1: *p* = 0.001, week 2: *p* = 0.019 and week 3: *p* = 0.036). During the following therapy weeks, the *p*-values remained quite small, in week eight the *p*-value became once again non-significantly larger (*p* = 0.180). Four-week time intervals confirm the result (Table 6).

**Table 3:** Fixed effects of measurement time in the linear mixed models for the general module.

Dependent Variable	Estimator	SE	Counter df	*p*	F	df	AIC	CAIC
Total Scale	0.025	0.01	1	<0.001	18.81	770.10	1920.70	1949.58
Motor functioning scale	0.025	0.01	1	<0.001	15.47	781.42	2126.03	2154.90
Mental functioning scale	0.031	0.01	1	<0.001	21.63	770.36	2237.50	2266.38
Psychosocial functioning scale	0.019	0.01	1	0.007	7.20	781.99	2274.671	2303.55

Assessments = 876, Assessors N = 30.

**Table 4:** Random effects of measurement time in the linear mixed models for the general module.

	Total Scale General Module		Motor Functioning Scale		Mental Functioning Scale		Psychosocial Functioning Scale	
Parameter	Estimator	*p*	Estimator	*p*	Estimator	*p*	Estimator	*p*
Variance of the constant term therapist	1.25	<0.001	1.94	0.003	2.04	0.018	1.23	0.021
Variance of the constant term patient	0.73	0.008	0.80	<0.001	1.44	<0.001	0.91	<0.001
Residual variance	0.35	<0.001	0.44	<0.001	0.48	<0.001	0.54	<0.001

Assessments = 876, Assessors N = 30.

**Table 5:** Changes in patients over time in the general module (aggr).

Changes over Time	Min	Max	M	SD
Total scale Start–End	−3.44	4.28	0.3	1.01
Total scale Min.–Max.	0.00	5.40	1.2	1.13
Motor functioning scale Start–End	−3.80	3.50	0.2	1.00
Motor functioning scale Min.–Max.	0.00	6.90	1.3	1.32
Mental functioning scale Start–End	−4.29	4.29	0.4	1.21
Mental functioning Scale Min.–Max.	0.00	5.43	1.4	1.28
Psychosocial functioning scale Start–End	−3.37	6.13	0.3	1.23
Psychosocia functioning scale Min.–Max.	0.00	6.63	1.4	1.31

N = 125, 15 assessment weeks.

**Table 6:** Changes in the general module of patients in four-week time intervals (aggr).

Changes over Time	n	Min	Max	M	SD
1–4 weeks start–end	123 *	−3.92	4.92	0.12	1.09
4 weeks Min.–Max.		0.00	5.40	0.81	1.00
5–8 weeks start–end	79 *	−2.52	2.84	0.33	0.74
8 weeks Min.– Max.		0.00	2.84	0.70	0.69
9–12 weeks start–end	48 *	−1.72	1.60	−0.67	0.66
12 weeks Min.–Max.		0.00	2.60	0.67	0.59

* 1–4 weeks: 381 assessments in total, 5–8 weeks: 249 assessments in total, 9–12 weeks: 159 assessments in total.

### 3.2. EAT in the Individual (IS) and in the Group setting (GS) Submodules 

For the submodules IS and GS, confirmatory factor analyses (CFA) were performed to determine whether each of the proposed two-dimensional factor structures of the EFA (IS: Scale 1: Specific mental functioning, Scale 2: Specific motor functioning; GS: Scale 1: Interpersonal functioning, Scale 2: Intrapersonal functioning) fit the data. Since sample sizes were smaller than in the general module, CFA model fitting indices did not provide such precise indications to fit the data as in the general module.

Table 7 indicates that the two-factor structure of the hypothesized model of EAT IS still needs improvement to fit the data. The factor loadings of the hypothesized model were acceptable according to the usual criteria of Brown, but the global fit statistics needed modification [15]. In the first step, an error correlation was added between Items HFPE5 and HFPE8 (HFPE05 <--> HFPE8, MI = 78), since both items represent different aspects of mental capacity. The factor loadings remained robust, while the fit indices improved slightly (see Model 2, Table A2, Appendix A). Modification indices showed no more indications for further model fitting. Estimates showed a low standardized regression weight loading for Item HFPE4 (MI = 44), so it was removed. Model 3 shows improvement in the global fit statistics (CFI = 0.908), but due to the small sample size, modification indices did not show any further indications on how to modify the model, and therefore Model 3 represents the best-fit model for the data (χ² = 132.3, df = 42, CFI ≤ 0.90, RMSEA = 0.137, AIC = 180.33, CAIC = 270.21, see Table 8).

#### 3.2.1. Hypothesized Model (Model 1) Submodule IS

**Table 7:** AMOS Output for Hypothesized Model: Goodness-of-Fit Statistics.

Baseline Comparisons				
RMSEA				
Model	CFI	RMSEA	LO 90	HI 90
Default model	0.867	0.151	0.128	0.174
Saturated model	1.000			
Independence model	0.000	0.370	0.351	0.389
**AIC**				
**Model**	**AIC**	**BCC**	**BIC**	**CAIC**
Default model	240.160	246.596	308.784	333.784
Saturated model	156.000	176.079	448.105	448.105
Independence model	1119.574	1122.663	1152.513	1164.513

#### 3.2.2. Final Model (Model 3) Submodule IS

**Table 8:** AMOS Output for Final Model: Goodness-of-Fit Statistics.

Baseline Comparisons				
RMSEA				
Model	CFI	RMSEA	LO 90	HI 90
Default model	0.908	0.137	0.111	0.164
Saturated model	1.000			
Independence model	0.000	0.396	0.376	0.418
**AIC**				
**Model**	**AIC**	**BCC**	**BIC**	**CAIC**
Default model	180.328	185.975	246.207	270.207
Saturated model	132.000	147.529	313.166	379.166
Independence model	1062.106	1064.694	1092.300	1103.300

The reliabilities of the scales of IS are in the good-to-excellent range (total scale α = 0.94; specific mental functioning scale α = 0.93; specific motor functioning scale α = 0.81), while the reliability of the total scale of the submodule is in the excellent range with α = 0.83. Due to the high reliability, a further reduction in the instrument is possible for an improved temporal–economic implementation. McDonald’s omega confirmed scores for the total scale (ω = 0.94) and subscale (specific mental functioning scale ω = 0.94). Omega for subscale specific motor functioning scale could not be executed due to the small number of items (2 Items). 

Spearman rank-correlation coefficients were calculated to estimate retest reliability because Q-Q diagrams and scatter plots did not show a normal distribution and linearity. Spearman tests showed significant values (total scale r = 0.488 **, *p* < 0.001; specific mental functioning scale r= 0.461 **, *p* < 0.001, specific motor functioning scale r= 0.526 **, *p* < 0.001, N = 52). 

For the measurement of inter-rater reliability, ICC showed significant values for time effects from the first until the third measurement (measurement time 1: ICC= 0.873, α = 0.895, *p* < 0.001; measurement time 2: ICC = 0.845, α = 0.855, *p* < 0.001, measurement time 3: ICC = 0.827, α = 0.840, *p* < 0.001, n = 10 assessed by three rater). A non-parametric Friedman test with Bonferroni correction showed significant values (Friedman-Test: Chi-Square (2) = 8.600, *p* = 0.014, n = 10). Pairwise comparison showed a significant change between the first and third measurements (*p* = 0.004). The change in values between the first and second and between the second and third measurements remained non-significant (*p* = 0.074, *p* = 0.264). The consistency of rater agreement also remained non-significant (ICC < 0, α = 0.079, *p* = 0.477; n = 10 assessed by three raters). 

The fixed effects of the linear mixed model for the total scale of the IS submodule and for both subscales show no significant change over time with respect to the measurement timepoint (total scale r = −0.001, *p* = 0.825; specific mental functioning scale r = −0.001, *p* = 0.586, specific motor functioning scale r = 0.010, *p* = 0.443). Table 9 and Table 10 show descriptive changes over time for the mixed linear model of submodule IS, based on the aggregated dataset. Calculations were based on a small data set; therefore, descriptive changes may not have been significantly verified (see Table 9).

As Table 11 shows, the hypothesized two-factor structure of the EAT GS submodule also needed improvement to fit the data. Factor loadings were also acceptable according to the usual criteria, but global fit statistics required modification [15]. In total, eight models were calculated to generate a model with an optimal fit for the data (see Table 12, final model).

#### 3.2.3. Hypothesized Model (Model 1) Submodule GS

**Table 11:** AMOS Output for Hypothesized Model: Goodness-of-Fit Statistics.

Baseline Comparisons				
RMSEA				
Model	CFI	RMSEA	LO 90	HI 90
Default model	0.851	0.166	0.142	0.191
Saturated model	1.000			
Independence model	0.000	0.391	0.371	0.411
**AIC**				
**Model**	**AIC**	**BCC**	**BIC**	**CAIC**
Default model	270.476	280.976	337.055	364.055
Saturated model	182.000	217.389	406.398	497.398
Independence model	1128.732	1133.787	1160.788	1173.788

#### 3.2.4. Final Model (Model 8) Submodule GS

**Table 12:** AMOS Output for Final Model: Goodness-of-Fit Statistics.

Baseline Comparisons				
RMSEA				
Model	CFI	RMSEA	LO 90	HI 90
Default model	0.964	0.096	0.057	0.132
Saturated model	1.000			
Independence model	0.000	0.418	0.394	0.442
**AIC**				
**Model**	**AIC**	**BCC**	**BIC**	**CAIC**
Default model	123.881	132.962	192.926	220.926
Saturated model	132.000	153.405	294.750	360.750
Independence model	902.346	905.913	929.471	940.471

As a first step in the model fitting process, an error correlation between items HFPG10 and HFPG11 was added, since both items thematize ICF aspects of interacting with others (Item correlation e11 <--> e12, MI = 21). Fit indices improved slightly; factor loadings remained robust (see Model 2, Table A3, Appendix A). Model 3 showed an item correlation for Items HFPG9 and HFPG10, since both also include ICF aspects of elementary interpersonal activities as appreciation and understanding (e10 <--> e11). Therefore, an error correlation was added, which dissolved in the further modeling process due to the reduction in item HFGP9. Model 4 shows improvement in the global fit statistics (CFI > 0.900), but still needs improvement. An error correlation was added between items HFPG7 and HFPG10 (Item correlation e8 <--> e11, MI = 19), because both items include exercises involving the horse. In model 5, MI showed item correlations for Items HFPG3 and HFPG5 (Item correlation e3 <--> e6, MI = 12). Both items include aspects of the understanding of social situations, so another error correlation was added. Model 6 showed further progress in the global model fit (CFI = 0.928) but MI indicated an item correlation for Items HFPG1 and HFPG12 (Item correlation e1 <--> e5, MI = 9). The correlation indicates that item content is related. Both items thematize different affective components of consciousness processes, so another error correlation was added (HFPG1= expressing wishes and needs, HFPG12 = making decisions and finding solutions). Model 7 showed item correlations between items HFPG7 and HFPG8 since both items include different aspects of interaction in relationships, so another error correlation was added (Item correlation e8 <--> e9, MI = 9). Model 8 showed a satisfactory global model fit (CFI = 0.949, see Table A3, Appendix A), and factor loadings also remained robust. Modification indices showed a cross-loading of Item HFPG 9 with the intrapersonal functioning scale (HFPG9 <--- interpersonal functioning scale MI = 5, HFPG9 <--- intrapersonal functioning scale MI = 5), which indicates that the item does not clearly discriminate between the scales. Therefore, Item HFPG 9 was removed. The model fit of Model 8 improved, but item HFPG 13 did not discriminate clearly between the scales, (cross-loading HFPG9 <--- interpersonal functioning scale MI= 6, intrapersonal functioning scale MI = 6), so it was removed to ensure that the final model was the best-fitting model. CFI increased slightly above the ideal value in the final model, but other global fit statistics improved in line with the target, so the reduction was considered appropriate. Model 8 (Table 12) represents the best-fitting model for the data (χ² = 67.9, df = 38, CFI = 0.964, RMSEA = 0.096, AIC = 123.88, CAIC = 220.93).

The reliabilities of the scales are in the good-to-excellent range (total scale α = 0.95; intrapersonal functioning scale α = 0.89; interpersonal scale α = 0.91). McDonald’s omega (ML) confirmed scores for the total scale (ω = 0.95) and subscales (intrapersonal functioning scale ω = 0.89; interpersonal scale ω = 0.91), and thereby also takes into the account factor loadings and error correlations of the model (Brown, 2015) [15]. The dataset for retest reliability was very small, the data did not show normal distribution and linearity (N = 17), and the Spearman rank-correlation coefficient did also not show significant values (total scale r = −0.254, *p* = 0.163; intrapersonal functioning scale r = 0.022, *p* = 0.466, interpersonal functioning scale r = 0.407, *p* = 0.053). 

ICC showed low values for time effects (measurement time 1: ICC < 0, α = 0.886, *p* = 0.475; measurement time 2: ICC = 0.492, α = 0.659, *p* = 0.176, measurement time 3: ICC < 0, α = −0.916, *p* = 0.783; n = 5 assessed by three raters). A normal distribution was not assumed due to the small sample size. A non-parametric Friedman test with Bonferroni adjustment showed significant results (Friedman-Test: Chi-Square (2) = 8.600, *p* = 0.015, n = 5). Pairwise comparison showed a significant change between the first and last measurements (*p* = 0.004). Rater agreement remained non-significant (ICC < 0, α > 0.999, *p* = 0.994; n = 5 assessed by three raters). 

The results of the linear mixed model for the GS submodule show a significant effect over time (total scale r = 0.038, *p* = 0.005; interpersonal functioning scale r = 0.044, *p* = 0.002, intrapersonal functioning scale r = 0.033, *p* = 0.033, see Table 13 and Table 14). This indicates that the submodule sensitively depicts change in functional ability over the course of therapy. A normal distribution test of the residuals confirms this for the total scale and both subscales. Table 15 and Table 16 show changes during therapy of the submodule GS over time, based on an aggregated dataset.

### 3.3. Hippotherapy Submodule (H)

For the submodule H, confirmatory factor analysis confirmed a two-dimensional factor structure based on previously calculated bivariate correlations on a different sample (Scale 1: Movement functioning, Scale 2: Motor control functioning). The sample size of the initial sample for EFA was too small to fulfill the requirements to execute EFA; therefore, bivariate correlation gave indications on the conceptual model structure. Table 17 indicates that the proposed conceptual model needs improvement to fit the data. According to Brown, the factor loadings were acceptable, while the global fit statistics needed modification [15]. In the first step, modification indices showed a residual correlation for Items H2 and H5 since both items represent mobility of body structures. Since both items represent different aspects of movement related functions, an error correlation was added and both items were retained (Model 2), (e7 <---> e8, MI= 32.8). Factor loadings remain robust. Fit indices improved slightly (see Model 2, Table A4, Appendix A). Furthermore, modification indices showed a residual correlation between Items H19 and H21 (e1 <--> e2 MI = 20.8) because both items thematize different aspects of the specific function “walking” as a movement-related aspect. As a result, an error correlation was added and Model 4 was run. Modification indices of model 4 indicate high cross-loadings of Item H22 with the movement functioning (MI = 6.7) and motor control functioning scales (MI = 6.8). Item H22 was therefore reduced to clearly distinguish the scales. In Model 5, global model fit increased, especially chi-squared (χ² = 240.772). Despite the increased model fit of Model 5, modification indices showed a residual correlation between Items H11 and H16 (e12 <--> e17 MI = 11.1), since both items thematize aspects of muscle activation; therefore, an error correlation was added, and the model was run again. Model 6 modification indices showed a residual correlation between Items H8 and H18 (e6 <--> e14 MI = 9.6), both of which thematize the range of movement functioning; therefore, an error correlation was added. Model 7 showed a noticeable improvement in model fit (CFI 0.906), but still needed improvement for a suitable data fit. Modification indices showed a high residual correlation for Items H7 and H19 (e2 <--> e11 MI = 8.5), since both items thematize functions of motion sequences responsible for movement patterns, and as such an error correlation was added. Model 8 made further progress in the global model fit (CFI = 0.913): modification indices showed a residual correlation between Items H10 and H23 (e4 <--> e16 MI = 7.9), and therefore an error correlation was added, and the model run again. The overall model fit of Model 9 was acceptable and represents the best fitting model for the data, including all parameters that are meaningful and relevant (χ² = 196.049, df = 96, CFI < 0.90, RMSEA = 0.133). The RMSEA could still be improved, since the limiting factor to further increasing the global model fit was the small sample size, which showed few modification indices, and would not improve global model fit of Model 9 significantly (N = 60). Therefore, to prevent overfitting of the model to this dataset, Model 9 represents the best model fit (see Table 18).

#### 3.3.1. Hypothesized Model (Model 1) Submodule H

**Table 17:** AMOS Output for Hypothesized Model: Goodness-of-Fit Statistics.

Baseline Comparisons				
RMSEA				
Model	CFI	RMSEA	LO 90	HI 90
Default model	0.776	0.208	0.187	0.230
Saturated model	1.000			
Independence model	0.000	0.410	0.391	0.429
**AIC**				
**Model**	**AIC**	**BCC**	**BIC**	**CAIC**
Default model	489.786	520.518	563.088	598.088
Saturated model	306.000	440.341	626.435	779.435
Independence model	1518.155	1533.082	1553.759	1570.759

#### 3.3.2. Final Model (Model 9) Submodule H

**Table 18:** AMOS Output for Final Model: Goodness-of-Fit Statistics.

Baseline Comparisons				
RMSEA				
Model	CFI	RMSEA	LO 90	HI 90
Default model	0.919	0.133	0.106	0.159
Saturated model	1.000			
Independence model	0.000	0.418	0.398	0.438
**AIC**				
**Model**	**AIC**	**BCC**	**BIC**	**CAIC**
Default model	276.049	308.430	359.823	399.823
Saturated model	272.000	382.095	556.831	692.831
Independence model	1389.707	1402.660	1423.217	1439.217

The reliabilities of the submodule H scales are in the excellent range (total scale α = 0.97; movement functioning scale α = 0.95; motor control functioning scale α = 0.94). Due to the high reliability, a further reduction in the instrument is possible for an improved temporal–economic implementation. McDonald’s omega (ML) confirmed scores for the total scale (ω = 0.97) and subscales (movement functioning scale ω =0.96; motor control functioning scale ω = 0.95), and via this procedure also considers the factor loadings and error correlations of the model. The dataset for retest reliability was too small to compute meaningful results for submodule H (N = 2). ICC showed significant values for time effects from the first to the third measurement (measurement time 1: ICC = 0.990, α = 0.998, *p* < 0.001; measurement time 2: ICC = 0.991, α = 0.997, *p* < 0.001, measurement time 3: ICC = 0.991, α = 0.998, *p* < 0.001, N = 3, assessed by three raters). A non-parametric Friedman test indicates no significant change during therapy (Chi-Square (2) = 2.000, *p* =0.368, n = 3). Multiple comparisons were not performed because the overall test resulted in the null hypothesis. Rater agreement also remained non-significant (ICC: 0.068, α = −0.073, *p* = 0.400; N = 3 assessed by three raters).

Concerning change sensitivity, the linear mixed model for the H submodule shows a significant effect over time in the movement functioning scale (r = 0.047, *p* = 0.009). The total scale and motor control functioning scale do not show significant time effects (total scale r = 0.023, *p* = 0.183; interpersonal functioning scale r = 0.002, *p* = 0.935) (Table 19 and Table 20). A normal distribution test of the residuals confirms the results for the total scale and subscales. Random effects for the total scale and the motor control functioning scale could not generate meaningful results due to the small sample size.

Table 21 and Table 22 show changes during therapy of submodule H over time, calculated using linear mixed models based on aggregated data. The means in Table 21 show that changes over time result in negative values (total scale start–end: M = −0.02, motor control functioning scale start–end: M = −0.13).

The developed assessment tool for the measurement of functioning in EAT contains 63 items in total (the complete tool can be found in Appendix B). The general module contains 25 items. Five items were reduced to ensure a targeted elaboration of the most economical model. The IS and GS submodules each contain 11 items. In the submodule IS, one item was dropped in the model fitting process. In the submodule GS, two items were dropped. Submodule H contains 16 items. Seven items of the submodule H were reduced based on content-related misfits in close consultation with therapist reviewers before the execution of model fit analyses. The submodule H contained more items than the other submodules, due to the absence of EFA modification indications in the pre-stage of this study. One item was reduced in the model fitting process to increase the global model fit statistics of Submodule H.

## 4. Discussion

In this study, the psychometric properties of the ICF-based digital assessment tool were analyzed via simultaneous confirmatory factor analyses (CFA) and reliability and sensitivity tests. The results of the general EAT module show that the three-factor model structure of the final model (Model 14) represents a suitable measure to assess the rehabilitation impacts of EAT. In further statistical research, short scales of the general module could be conducted to reduce items with error correlations. Alternatively, a more complex equivalent model (bifactor model) could be developed for the error-correlated items to ensure a more differentiated item structure. Regarding internal validity and plausibility, the item bank of the general EAT model proves itself to be discriminant in the proposed three-factor structure model fit via the simultaneous CFA. Convergent validity is given because of high factor loadings on the proposed factors. Divergent validity is given by the correlations between the factors, which is significantly different from 1.0; therefore, the factors measure clearly distinguishable aspects of patient functioning in EAT interventions. 

Multi-level modelling confirmed the change sensitivity of the module. Retest reliability proved test stability over time. The rater agreement of the therapists was generally in need of improvement for the main module and all submodules. The rater variance of the therapists in the functional measurement can be explained on the one hand by the general error-proneness of rating scales due to typical judgment errors (e.g., strict-mild errors or halo effect), and on the other hand by the absence of a consistent recurring test situation in therapeutic practice to constantly survey specific performance characteristics [16]. Furthermore, therapists did not know the assessed patients and their medical history. A study by Cronley, Marchant, and Caldarella showed for teachers that their assessments were highly reliable in estimating the frequency of occurrence of behavior problems in pupils when the teachers knew how these behaviors presented themselves [16,17]. Accordingly, the assessing therapists should be intensively involved in the individual therapy to conduct assessments that are more reliable. However, this increases the risk of detection bias, in which a therapist’s own intervention may be assessed as more effective because the therapist is not blinded and is convinced of the effectiveness of their intervention. 

Additionally, for the assessment of hippotherapy, seasonal influences were a cause of variance, since participants wore jackets in winter, which made the assessment of their physical functioning status particularly difficult to accurately assess. The 10-step Likert scale represents another cause of variance, given that too many response categories can negatively affect the measurement properties of the items since the high degree of differentiation can overburden assessors [13]. Aside from reasons of the measurability of change sensitivity, the 10-step scale represented the most appropriate one for the developed assessment tool, since a more differentiated response format provides more opportunities to make distinctions between patients [13]. A promising approach for future research would be the test for inter-rater reliability using generalizability theory [16,18]. According to this theory, the causes of measurement errors can be estimated and their conditions simulated, so that relative and absolute comparisons of progress-diagnostics can be examined precisely [16,19]. Thus, according to the numerical invariance concept, it would not be problematic that therapeutic professionals do not assess identically as long as they assess trends in the same way, which is more realistic in behavioral observations over time [16,18]. 

Causes for negative and non-significant values in the submodule analyses of therapy progress over time can also be explained by the functional capacities of the therapies’ target groups. Patients with chronic degenerative diseases or multiple disabilities may face mental and motor functioning decreases over time (for example patients with multiple sclerosis). In these contexts, therapies succeed by delaying the degenerative progression of the diseases. An improvement in functioning cannot always be achieved in therapy and therefore cannot be detected by assessment tools. Assessed patients in the ICC for submodule H were diagnosed with Huntington’s chorea, and Angelman’s syndrome; where maintaining the current functional state can already represent therapeutic success for EAT. A study by Goudy et al. (2019) came to similar conclusions. Positive effects of an hippotherapy simulator program for older adults with Parkinson’s Syndrome were found with regard to an increase in balance and cognitive impairment [20]. The authors argue in favor of slowing the natural progression of the disease by improving symptoms such as an increased balance and posture through these types of interventions [20]. Fizkova et al. (2013) point out that hippotherapy is effective in suppressing pathological stereotypy of muscle groups and promotes postural reflex mechanisms in children with cerebral palsy [21]. Vermöhlen et al. (2017) showed positive effects of hippotherapy, alongside treatment as usual, in the improvement of balance, fatigue, spasticity, and quality of life in patients with multiple sclerosis [22]. EAT interventions aim to influence components of balance and motor control, which then promote motor problem solving skills that improve ambulation and sitting and other life-related activities [23]. Furthermore, positive effects in regard to post-traumatic stress disorders (PTSD) and psychosocial functioning have been found by Johnson et al. (2018) and Gabriels et al., 2015 [24,25]. The study results discussed thus far show that these therapies are effective, but that instruments may not be accurately and adequately targeting their holistic therapy approaches. Researchers furthermore emphasize that the field of EAT would benefit from future research testing different assessment tools and intervention protocols to precisely assess therapy effects and evaluate therapeutic outcomes [23]. In this respect, the general module of the assessment tool satisfactorily fulfills psychometric requirements and can directly be applied in therapeutic practice. 

Overall, the developed digital assessment tool represents a standardized ICF application suitable for targeted assessment of functioning in EAT. As previous studies indicate, the ICF defines a promising framework for the identification of beneficial aspects of holistic EAT interventions on human functioning combined with the possibility of quantification of therapy effects. Therefore, the assessment tool developed and validated herein contributes to the joint efforts of the international scientific community to increase evidence of the effects of EAT in international healthcare through systematic assessment strategies [5,6,7]. The assessment tool can sensitively measure therapy progress change and also precisely depict the effect factors of the therapy.

For the submodules, the small sample sizes for performing CFA and reliability and change sensitivity tests were a limiting factor. The final CFA models of the submodules IS and GS still show deviating values in the global fit statistics, while modification indices did not provide further options to increase model fit. Test stability (retest and inter-rater reliability) could not be estimated in a target-oriented manner to depict significant effects. A cause of the small sample sizes was patient acquisition problems based on restrictions due to the COVID-19 pandemic in Germany during the data collection period from August 2020–2021. Further research should determine reliability scores based on larger sample sizes. In respect to reliability, the possibility of distortive effects based on the assessments carried out by EAT therapists can also not be ruled out. In the future, the developed ICF-based assessment tool could be trialed in a controlled randomized study with a group of patients homogeneous with respect to age, gender, and disability or chronic disease. Thus, the effects of the EAT interventions and conclusions about reliability could be assessed in a more precise and differentiated manner. 

Regarding process orientation, the developed assessment tool builds the basis to monitor rehabilitation impacts and guide the therapy progression of EAT within the WHO and ICF frameworks, which represent the international standard of global health systems. As such, it refers to the innovative resource-orientated functioning approach, which operationalizes a person’s “lived health” in addition to the biological health status to perform activities and participate in social situations. In the future, this assessment tool could be used in close coordination with physicians and multi-professional rehabilitation teams for a targeted overall rehabilitation process and electronic data interchange within institutions [26]. Furthermore, for goal setting with clients, the assessment tool could be a promising instrument to align therapy goals related to desired life skills, as other studies indicate [26]. Past research has shown that the implementation of the ICF in the rehabilitation team had a positive impact that manifested in a more systematic work approach, greater interdisciplinary cooperation, and a participatory orientation in the clinical setting [26]. In addition, for funding agencies and stakeholders involved in the rehabilitation process, transparent planning of rehabilitative processes and services via the ICF could provide a better insight into processes and their financial aspects, which may lead to increased cost-reimbursements from public financiers and insurance companies [23,26]. For the field of EAT, the prospective usage of the developed assessment tool builds a basis for increased comparison and joint collection of EAT findings across languages and countries [27]. It could encourage the usage of a common international terminology based on the WHO language and thereby help incorporate EAT into global health systems. Therapy contents and outcomes could be assessed more precisely and linked back into the ICF-classification system to help gather scientific evidence about EAT effect factors through international collaboration. 

## 5. Conclusions

The validated assessment tool provides a nuanced framework for evaluating the therapy outcomes and effect factors of EAT interventions in the common language of the ICF. Through its connection to concrete ICF categories, EAT effects prospectively could be evaluated in a more standardized manner in multicultural and multi-professional health care teams. Therapy effects could also be compared to other functioning-related intervention outcomes in international health systems. This could enable economic cost-effectiveness evaluations and therefore affect a targeted outcome measurement in the context of formal therapy evaluations based on functioning capabilities. Furthermore, common international efforts to create scientific databases with large-scale data from multicenter studies and international research collaborations could be implemented in a targeted manner through unified study variables and specific therapy insights based on ICF parameters.

## 6. Patents

A legally protected trademark is declared in 2022 by Research Institute for Inclusion through Physical Activity and Sport. Further information can be obtained from the authors.

## Figures and Tables

**Table 2:** AMOS Output for Final Model: Goodness-of-Fit Statistics.

Baseline Comparisons				
RMSEA				
Model	CFI	RMSEA	LO 90	HI 90
Default model	0.914	0.090	0.083	0.097
Saturated model	1.000			
Independence model	0.000	0.286	0.280	0.292
**AIC**				
**Model**	**AIC**	**BCC**	**BIC**	**CAIC**
Default model	945.928	959.256	1164.291	1225.291
Saturated model	650.000	721.008	1813.412	2138.412
Independence model	6831.792	6837.254	6921.285	6946.285

**Table 9:** Changes in submodule IS over time.

Changes over Time	Min	Max	M	SD
total scale start–end	−2.18	1.73	−0.01	0.79
total scale Min.–Max.	0.00	2.64	0.92	0.82
Specific mental functioning scale start–end	−2.11	2.00	−0.3	0.85
Specific mental functioning scale Min.–Max.	0.00	2.78	0.92	1.28
Specific motor functioning scale start–end	−2.50	2.50	0.71	0.96
Specific motor functioning scale Min.–Max.	0.00	4.50	1.4	1.30

**Table 10:** Changes in submodule IS with four-week therapy periods.

Changes over Time	n	Min	Max	M	SD
1–4 weeks start–end	179	−2.45	1.64	−0.05	0.80
4 weeks Min.–Max.	179	0.00	2.45	0.05	0.67
5–8 weeks start–end	105	−1.73	1.27	−0.10	0.63
8 weeks Min.–Max.	105	0.00	2.09	0.73	0.46
9–12 weeks start–end	83	−1.73	0.82	−0.15	0.62
12 weeks Min.–Max.	83	0.00	1.73	0.06	0.52

**Table 13:** Fixed effects of the linear mixed models for the total scale of the submodule GS and both subscales with relation to measurement time points.

Dependent Variable	Estimator	SE	Counter df	*p*	F	df	AIC	CAIC
Total scale	0.038	0.01	1	0.005	7.86	347.447	1021.215	1045.929
Interpersonal functioning scale	0.044	0.01	1	0.002	9.49	348.601	1064.704	1089.418
Intrapersonal functioning scale	0.033	0.01	1	0.019	5.51	347.687	1046.401	1070.955

Assessments = 381, (9 assessors).

**Table 14:** Random effects of the linear mixed model for the total scale and subscales submodule GS.

Dependent Variables	Estimator	*p*
Total scale variance of the constant term therapist	0.21	0.288
Total scale variance of the constant term therapist*patient	0.67	<0.000
Residual variance	0.60	<0.001
Interpersonal functioning scale variance of the constant term therapist	0.16	0.350
Interpersonal functioning scale variance of the constant term therapist*patient	0.70	<0.001
Residual variance	0.69	<0.000
Intrapersonal functioning scale variance of the constant term therapist	0.24	0.279
Intrapersonal functioning scale variance of the constant term therapist*patient	0.72	<0.000
Residual variance	0.64	<0.000

**Table 15:** Changes in submodule GS over time.

Changes over Time	Min	Max	M	SD
total scale start–end	−2.18	4.64	0.53	1.30
total scale Min.–Max.	0.00	5.36	1.48	1.37
Interpersonal functioning scale start–end	−2.60	4.80	0.60	1.38
Interpersonal functioning scale Min.–Max.	0.00	5.80	0.92	1.43
Intrapersonal functioning scale start–end	−2.00	4.50	0.47	1.30
Intrapersonal functioning scale Min.–Max.	0.00	5.50	1.51	1.41

**Table 16:** Changes in submodule GS in the context of four-week therapy periods.

Changes over Time	*n*	Min	Max	M	SD
1–4 weeks start–end	205	−2.36	5.36	0.38	1.58
4 weeks Min.–Max.	205	0.00	5.36	1.24	1.30
5–8 weeks start–end	123	−2.36	2.09	0.13	1.00
8 weeks Min.–Max.	123	0.00	2.36	1.10	0.70
9–12 weeks start–end	49	−0.91	2.73	0.36	0.96
12 weeks Min.–Max.	49	0.00	2.73	0.91	0.74

**Table 19:** Fixed effects of the linear mixed models for the total scale of the submodule H and both subscales with relation to measurement time points.

Dependent Variable	Estimator	SE	Counter df	*p*	F	df	AIC	CAIC
Total Scale	0.023	0.02	42.5	0.183	68.2	10.076	122.939	137.695
Movement functioning scale	0.047	0.02	42.4	0.009	7.6	42.364	125.568	140.325
Motor control functioning scale	−0.002	0.02	42.7	0.935	0.0	42.694	136.022	150.779

Assessments = 52, (9 assessors, 10 patients).

**Table 20:** Random effects of the linear mixed model for the total scale and subscales, submodule H.

Dependent Variables	Estimator	*p*
Total scale variance of the constant term therapist	0.00 #	#
Total scale variance of the constant term therapist*patient	5.13	0.031
Residual variance	0.24	<0.001
Movement functioning scale variance of the constant term therapist	0.09	0.987
Movement functioning scale variance of the constant term therapist*patient	5.90	0.359
Residual variance	0.25	<0.001
Motor control functioning scale variance of the constant term therapist	0.00 #	#
Motor control functioning scale variance of the constant term therapist*patient	4.72	0.034
Residual variance	0.34	<0.001

# Could not be calculated.

**Table 21:** Changes in submodule H over time.

Changes over Time	Min	Max	M	SD
total scale start–end	−1.75	1.13	−0.02	0.71
total scale Min.–Max.	0.00	2.06	0.48	0.79
Movement functioning scale start–end	−1.12	1.75	0.09	0.69
Movement functioning scale Min.–Max.	0.00	2.25	0.51	0.85
Motor control functioning scale start–end	−2.37	0.63	−0.13	0.82
Motor control functioning scale Min.–Max.	0.00	2.63	0.53	0.92

**Table 22:** Changes in submodule H with four-week therapy periods.

Changes over Time	n	Min	Max	M	SD
1–4 weeks start–end	19	−0.94	0.00	−0.37	0.38
4 weeks Min.–Max.	19	0.00	2.06	0.80	0.18
5–8 weeks start–end	12	−1.19	0.94	−0.31	0.95
8 weeks Min.–Max.	12	0.69	1.19	0.94	0.21
9–12 weeks start–end	12	0.00	0.13	0.05	0.06
12 weeks Min.–Max.	12	0.00	0.19	0.06	0.09

## Data Availability

Data can be obtained from the authors.

## Appendix A. Details of the CFA Process

**Table A1:** Goodness-of-Fit statistics Module G.

*Robust Fit Indices for 14 Proposed Models for the General EAT Module (G)*							
Goodness-of-Fit Indices							
Model	χ²	df	PCMIN/DF	CFI	RMSEA	AIC	CAIC
Model 1 (Hypothesized Model)	1976.6	402	4.917	0.810	0.122	2102.63	2391.2
Model 2 (-Item G1)	1766.6	374	4.728	0.825	0.119	1888.6	2167.9
Model 3 (+ correlated error e13 <--> e14)	1675.2	373	4.493	0.836	0.115	1799.7	2083.6
Model 4 (+ correlated error e4 <--> e5)	1592.4	372	4.280	0.847	0.111	1718.40	2006.9
Model 5 (-Item G14)	1405.8	345	4.074	0.853	0.110	1522.77	1852.1
Model 6 (-Item G11)	1252.1	319	3.925	0.868	0.105	1370.07	1640.3
Model 7 (+ correlated error e2 <--> e3)	1204.9	318	3.777	0.875	0.103	1324.90	1599.7
Model 8 (+ correlated error e26 <--> e27)	1166.5	317	3.679	0.880	0.101	1288.48	1567.8
Model 9 (-Item G2)	1063.	292	3.640	0.887	0.100	1180.96	1451.2
Model 10 (-Item G18)	959.3	268	3.579	0.893	0.099	1073.32	1334.4
Model 11 (+ correlated error 3 <--> e4)	928.6	267	3.477	0.898	0.097	1044.60	1310.2
Model 12 (+ correlated error e5 <--> e6)	901.0	266	3.387	0.902	0.095	1019.04	1289.2
Model 13 (+ correlated error e4 <--> e6)	847.0	265	3.196	0.910	0.091	966.97	1241.8
*Model 14* *(+ correlated error e25 <--> e26)* *(Final Model)*	*823.9*	*264*	*3.120*	*0.914*	*0.090*	*945.93*	*1225.3*

**Table A2:** Goodness-of-Fit statistics Submodule IS.

*Robust Fit Indices for 3 Proposed Models for the EAT Individual Submodule IS*							
Goodness-of-Fit Indices							
Model	χ²	df	PCMIN/DF	CFI	RMSEA	AIC	CAIC
Model 1 (Hypothesized Model)	190.160	53	3.588	0.867	0.151	240.160	333.784
Model 2 (+ error correlation Items HFPE5 & HFPE8)	166.081	52	3.194	0.889	0.139	218.081	315.449
*Model 3* *(-Item HFPE 4)*	*132.328*	*42*	*3.151*	*0.908*	*0.137*	*180.328*	*270.207*

**Table A3:** Goodness-of-Fit statistics for Submodule GS.

*Robust Fit Indices for Nine Proposed Models for the EAT GROUP Submodule GS*							
Goodness-of-Fit Indices							
Model	χ²	df	PCMIN/DF	CFI	RMSEA	AIC	CAIC
Model 1 (Hypothesized Model)	216.476	64	3.382	0.851	0.166	270.476	364.055
Model 2 (Item correlation e11 <--> e12)	191.748	63	3.044	0.874	0.154	247.748	344.793
Model 3 (Item correlation e10 <--> e11)	182.522	62	2.944	0.882	0.150	240.522	341.034
Model 4 (Item correlation e8 <--> e11)	159.104	61	2.608	0.904	0.137	219.104	323.081
Model 5 (Item correlation e3 <--> e6)	146.404	60	2.440	0.916	0.129	208.404	315.847
Model 6 (Item correlation e1 <--> e5)	132.924	59	2.253	0.928	0.121	196.924	307.834
Model 7 (Item correlation e8 <--> e9)	123.252	58	2.125	0.936	0.114	189.252	303.627
Model 8 (-Item HFPG 9)	95.126	48	1.982	0.949	0.107	155.126	259.104
*Model 9* *(-Item HFPG 13)*	*67.881*	*38*	*1.786*	*0.964*	*0.096*	*123.881*	*220.926*

**Table A4:** Goodness-of-Fit statistics Submodule H.

*Robust Fit Indices for 9 Proposed Models for Submodule H*							
Goodness-of-Fit Indices							
Model	χ²	df	PCMIN/DF	CFI	RMSEA	AIC	CAIC
Model 1 (Hypothesized Model)	419.8	118	3.558	0.776	0.208	489.8	598.1
Model 2 (+ correlated error e7 <--> e8)	372.2	117	3.181	0.811	0.192	444.3	555.6
Model 3 (+ correlated error e9 <--> e10)	326.5	116	2.814	0.844	0.175	400.5	515.0
Model 4 (+ correlated error e1 <--> e2)	301.4	115	2.621	0.862	0.166	377.4	495.0
Model 5 (-Item H22)	240.8	100	2.408	0.886	0.154	312.8	424.2
Model 6 (+ correlated error e12 <--> e17)	225.6	99	2.279	0.898	0.147	299.6	414.1
Model 7 (+ correlated error e6 <--> e14)	213.9	98	2.183	0.906	0.142	289.9	407.5
Model 8 (+ correlated error e2 <--> e11)	204.5	97	2.109	0.913	0.137	282.5	403.2
*Model 9* *(+ correlated error e4 <--> e16)*	*196.0*	*96*	*2.042*	*0.919*	*0.133*	*276.0*	*399.8*

## Appendix B

**Table A5:** Descriptive statistics of the ICF-based assessment tool (general EAT module and all submodules).

Items General Module	ICF Code	N	Min	Max	Mean	±SD
G03. Can establish a stabilized state of mind	b1263 Psychic stability	265	1	10	6.17	2.070
G04. Shows motivation	b1301 Motivation	265	1	10	7.78	1.943
G05. Can express their own needs	b130 Energy and drive functions	265	1	10	6.60	2.259
G06. Is able to realistically assess their own abilities	b1800 Experience of self	265	1	10	5.29	2.257
G07. Is able to achieve intentions and goals through planned actions	b1641 Organizing and planning	265	1	10	6.09	2.276
G08. Is able to adapt to new things or to face new experiences positively	b1264 Openness to experience	265	1	10	6.23	2.169
G09. Is able to build trust in others	b122 Global psychosocial functions	265	2	10	6.99	2.104
G10. Is able to regulate his/her feelings adequately in different situations. This includes dealing with anger or frustration	b1521 Regulation of emotion	265	1	10	5.54	2.181
G12. Can build and maintain a trusting relationship with the therapist	d7200 Forming relationships	265	1	10	7.48	1.909
G13. Can build and maintain a trusting relationship with the horse	d7200 Forming relationships	265	1	10	7.46	2.015
G15. Can show consideration and tolerance and react to the same	d7102 Tolerance in relationships	265	1	10	5.92	2.417
G16. Can engage in an activity for a period of time. The person/child is not distracted	b1400 Sustaining attention	265	1	10	5.85	2.574
G17. Can memorize processes and tasks in the therapy and reproduce them later	b1442 Retrieval of Memory	265	1	10	6.44	2.475
G19. Can understand the meaning of various facial expressions or nonverbal communication	d3150 Communicating with—receiving—body gestures	265	1	10	6.73	2.197
G20. Can express herself/himself in a communicative way	d330 Speaking	265	1	10	6.60	2.585
G21. Is able to stand up actively on horseback during movement	d4106 Shifting the body’s center of gravity	265	1	10	8.56	1.940
G22. Can keep their head upright and move it in a controlled manner	b760 Control of voluntary movement functions	265	1	10	7.46	2.294
G23. Is able to keep their balance while sitting on the horseback in motion	b235 Vestibular functions	265	1	10	7.19	2.174
G24. Can feel vibrations on the horse’s back	b2701 Sensitivity to vibration	265	1	10	7.19	1.953
G25. Can adjust their movements to a rhythm or adapt to it in an appropriate way, e.g., swing with the movement of the horse	b156 Perception functions	265	1	10	6.14	2.221
G26. Can control the tension of their muscles in a targeted manner	b7356 Tone of all muscles of the body	265	1	10	5.78	2.285
G27. Can perform a gross motor movement task in a targeted manner	b789 Functions of movement, unspecified	265	1	10	6.64	2.323
G28. Can perform a fine motor movement task in a targeted manner	d440 Fine hand use	265	1	10	5.69	2.354
G29. Can use both halves of the body as a complete system. This includes balancing physical asymmetries	b735 Muscle tone functions	265	1	10	6.11	2.367
G30. Shows a fluid movement pattern when performing movement tasks. This includes a dynamic, spatio-temporally correct movement sequence of coordinated partial movements	b799 Neuro-musculoskeletal and movement-related functions, unspecified	265	1	10	5.56	2.449
**Submodule IS**						
IS01. Is able to act in a thoughtful manner	b1644 Insight	115	1	10	6.04	2.367
IS02. Is able to exert a targeted and measured force according to a simple movement task	b7306 Power of all muscles of the body	115	1	10	5.68	2.134
IS03. Is able to perceive visual stimuli (this includes distinguishing shape, size, color, and other visual stimuli)	b1561 Visual perception	115	2	10	7.87	1.931
IS05. Is able to adapt their movements to the movements of the horse in a targeted manner	b1471 Quality of psychomotor functions	115	1	10	5.76	2.223
IS06. Is able to establish physical contact with the horse to an appropriate degree and react to it	d799 Interpersonal interactions and relationships, unspecified	115	1	10	6.04	2.265
IS07. Can maintain physical distance between him/herself and others	d7204 Maintaining social space	115	1	10	6.45	2.344
IS08. Can actively work toward achieving their personal goals	b164 Higher-level cognitive functions	115	1	10	5.30	2.359
IS09. Can stand up for him/herself	b130 Psychic energy and drive	115	1	10	5.35	2.410
IS10. Is able to control their actions appropriately in regard to the situation, e.g., remain calm in the presence of the horse	d7202 Regulating behaviors within interactions	115	1	10	6.11	2.445
IS11. Can overcome capriciousness and constantly changing moods	b1521 Regulation of emotion	115	1	10	5.51	2.249
IS12. Can independently find solutions to a question or situation	d175 Solving problems	115	1	10	5.17	2.583
**Submodule GS**						
GS01. Is able to express their own wishes and feelings	b130 Psychic energy and drive	87	2	10	6.13	1.946
GS02. Shows self-confidence	1266 Confidence	87	1	10	5.72	1.897
GS03. Understands the situation in dealing with the horse and acts in a thoughtful manner	b1644 Insight	87	2	10	6.17	1.760
GS04. Can take care of their physical and mental well-being in a way appropriate to his/her age	d570 Looking after one’s health	87	2	9	5.46	1.797
GS05. Can use and understand social signs such as gestures and facial expressions	d7104 Social cues in relationships	87	2	10	6.29	1.910
GS06. Can handle conflict constructively	d7103 Criticism in relationships	87	1	10	5.29	1.880
GS07. Is able to make physical contact with others and react to them, e.g., contact with the horse or sitting on the horse in pairs and doing an exercise together	d799 Interpersonal interactions and relationships, unspecified	87	1	10	6.32	1.908
GS08. Is able to establish and maintain relationships with others	d7200 Forming relationships	87	2	10	6.13	1.648
GS10. Can show consideration and appreciation for others or the horse and react to them	d7101 Appreciation in relationships	87	2	10	6.68	1.808
GS11. Can show understanding and acceptance toward behavior of others or the horse and respond to them	d7100 Respect and warmth in relationships	87	2	10	6.47	1.758
GS12. Can find solutions to problems or decisions in interaction with others or the horse	d175 Solving problems	87	1	10	5.55	1.921
**Submodule H**						
H02. Has functionally impaired joints that are mobilized and centered Example: The person/child can take up the physically correct position on the rider’s seat	b7100 Mobility of a single joint	60	1	10	7.53	2.639
H03. Can perceive position and alignment of individual parts of the body	b1470 Psychomotor control	60	1	10	6.07	2.680
H04. Can perceive proprioceptive stimuli (this includes, for example, the perception of movement and position)	b260 Proprioception function	60	1	10	6.27	2.510
H05. Has a fully mobile range of motion in their spine	b7100 Mobility of a single joint	60	1	10	7.23	2.788
H07. Can specifically control the speed of movement in sequences of motions	b1470 Psychomotor control	60	1	10	5.67	2.921
H08. Does not have a restricted total range of movement when performing movements	b710 Mobility of joint functions	60	1	10	6.13	3.306
H09. Has motion that is continuously fluid	b1470 Psychomotor control	60	1	10	5.27	2.916
H10. Has stable torso muscles	b7305 Power of muscles of the trunk	60	1	10	6.63	2.591
H11. Is able to keep their balance when sitting freely on a chair without support	d4153Maintaining a sitting position	60	1	10	7.68	3.332
H14. Can change the position of their body independently, e.g., moving from one place to another or standing up from a chair	d420 Transferring oneself	60	1	10	6.97	3.751
H16. Can regulate the muscle tone of their limbs in a targeted manner	b7354 Tone of muscles of all limbs	60	1	10	5.47	3.143
H17. Can perform a targeted movement of the lower limbs or the upper limbs without any associated movement	b7602 Coordination of voluntary movements	60	1	10	6.38	3.315
H18. Can adapt their motor movement behavior to the situation, e.g., respond to a sudden movement, such as when the horse stops	b755 Involuntary movement reaction functions	60	1	10	5.77	2.813
H19. Is able to walk unrestrictedly over short distances (approx. 50 m) without assistance or a break	d4500 Walking short distances	60	1	10	6.17	4.267
H21. Can independently negotiate inclines and declines, e.g., go up or down stairs or ramps	d4502 Walking on different surfaces	60	1	10	5.90	3.861
H23. Can control jaw and swallowing movements in a targeted manner	b1470 Psychomotor control	60	2	10	8.30	2.553

Note: Items were directly translated from the validated German version of the assessment tool with minor corrections.
